# Divergent karyotypes of the annual killifish genus *Nothobranchius* (Cyprinodontiformes, Nothobranchiidae)

**DOI:** 10.3897/CompCytogen.v10i3.9863

**Published:** 2016-09-16

**Authors:** Eugene Krysanov, Tatiana Demidova, Bela Nagy

**Affiliations:** 1 Severtsov Institute of Ecology and Evolution, Russian Academy of Sciences, Leninsky prospect, Moscow, 119071 Russia; 2 30, rue du Mont Ussy, 77300 Fontainebleau, France

**Keywords:** Africa, chromosome number, karyotype, killifish, Nothobranchius

## Abstract

Karyotypes of two species of the African annual killifish genus *Nothobranchius* Peters, 1868, *Nothobranchius brieni* Poll, 1938 and *Nothobranchius* sp. from Kasenga (D.R. Congo) are described. Both species displayed diploid chromosome number 2n = 49/50 for males and females respectively with multiple-sex chromosome system type X_1_X_2_Y/X_1_X_1_X_2_X_2_. The karyotypes of studied species are considerably different from those previously reported for the genus *Nothobranchius* and similar to the Actinopterygii conservative karyotype.

## Introduction

Annual killifishes belonging to the genus *Nothobranchius* Peters, 1868 are mainly distributed in eastern Africa but several species are found in central Africa (Wildekamp 2004). They inhabit temporary pools that dry out during the dry season and have specific adaptations for extreme environments. Annual fishes are characterised by specific life history traits of extremely short lifespan and diapause in embryonic development (Furness 2015, Nagy 2015). Their unique biology makes them a model taxon with which to investigate aging, embryonic development, ecology, and natural selection (Cellerino et al. 2015).

Killifishes of the genus *Nothobranchius* comprise 71 valid species (FishBase 2015). In this genus karyologicaly were described only 23 species (Arai 2011). These species have variable karyotypes with diploid chromosome numbers (2n) ranging from 2n = 16 for *Nothobranchius rachovii* Ahl, 1926 to 2n = 43 for *Nothobranchius thierryi* (Ahl, 1924) (Scheel 1990). More than 60% of karyotypes in *Nothobranchius* are characterised by a modal diploid number of 2n = 36-38.

A multiple-sex chromosome system of X_1_X_1_X_2_X_2_/X_1_X_2_Y type has been reported for only one species of *Nothobranchius*, *Nothobranchius guentheri* (Pfeffer, 1893) with a female karyotype consisting of 36 chromosomes and the male karyotype consisting of 35 chromosomes (Ewulonu et al. 1985).

In this paper, the karyotypes of two species, *Nothobranchius brieni* Poll, 1938 and *Nothobranchius* sp. from Kasenga, were studied, bringing the number of species studied to 25.

## Material and methods

**Figure 1.**
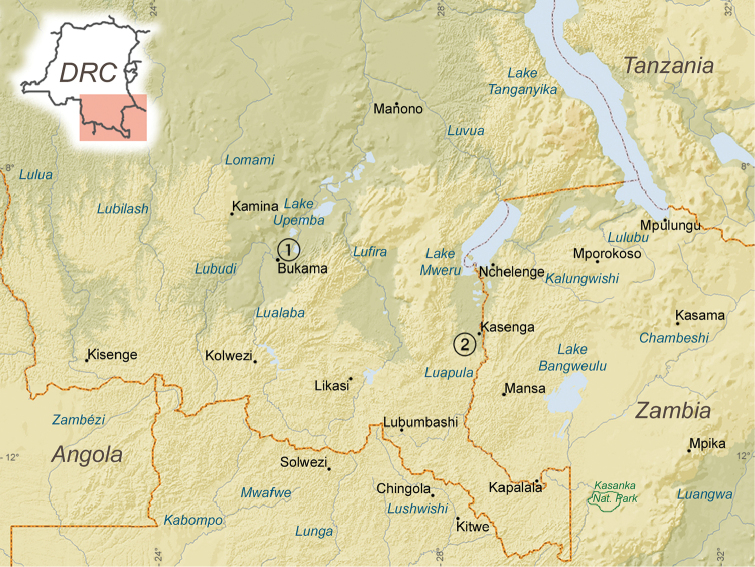
Localities of specimen collections in the Democratic Republic of Congo (**1** *Nothobranchius brieni* **2** *Nothobranchius* sp. ‘Kasenga’).

Specimens of *Nothobranchius brieni* were collected from a large ephemeral swamp in the Lualaba drainage, near the village of Bukama in Katanga province (Democratic Republic of Congo, 09°11.374'S 25°51.334'E) on 2 April 2013 by E. Abwe, B. Katemo Manda, and B. Nagy, whereas specimens of *Nothobranchius* sp. from Kasenga (*Nothobranchius* sp. ‘Kasenga’) were collected in an ephemeral swamp in the Luapula drainage, near Kasenga, a village in Katanga province (D.R. Congo, 10°31.360'S, 28°27.368'E) on 17 April 2015, by E. Abwe, A. Chocha Manda, B. Katemo Manda, and T. Popp (Fig. 1).

### Cytogenetic analysis

Chromosomes were prepared according to the Kligerman and Bloom method (1974). The chromosome preparations were obtained from head kidney tissue. Before preparation fish were treated intraperitoneally with 0.1% colchicine for 3–4 hours. The hypotonisation lasted 20–30 min at room temperature in 0.075 M KCl. Then tissue samples were fixed in 3:1 methanol : acetic acid for 24 hours. Six specimens of *Nothobranchius brieni* (three males and three females) and three specimens of *Nothobranchius* sp. ‘Kasenga’ (one male and three females) were karyotyped with this method. Meiotic chromosome preparations of *Nothobranchius brieni* were acquired from testes by the same technique.

Slides were dried by air and stained with 2% Giemsa solution in phosphate buffer at pH 6.8 for 10 min. Karyotypes were analysed under microscope “AxioImager” Karl Zeiss (Germany) equipped with CCD camera and “KaryoImage” Metasystems Software (Germany). In each specimen the chromosome number and type was determined on metaphase plate. Chromosome morphology was determined according to Levan et al. (1964). The chromosomes were classified as metacentric (M), submetacentric (SM), and acrocentric (A). To determine the fundamental number (NF), chromosomes of the M and SM groups were considered bi-armed and those of group A as uni-armed.

## Results

**Figure 2.**
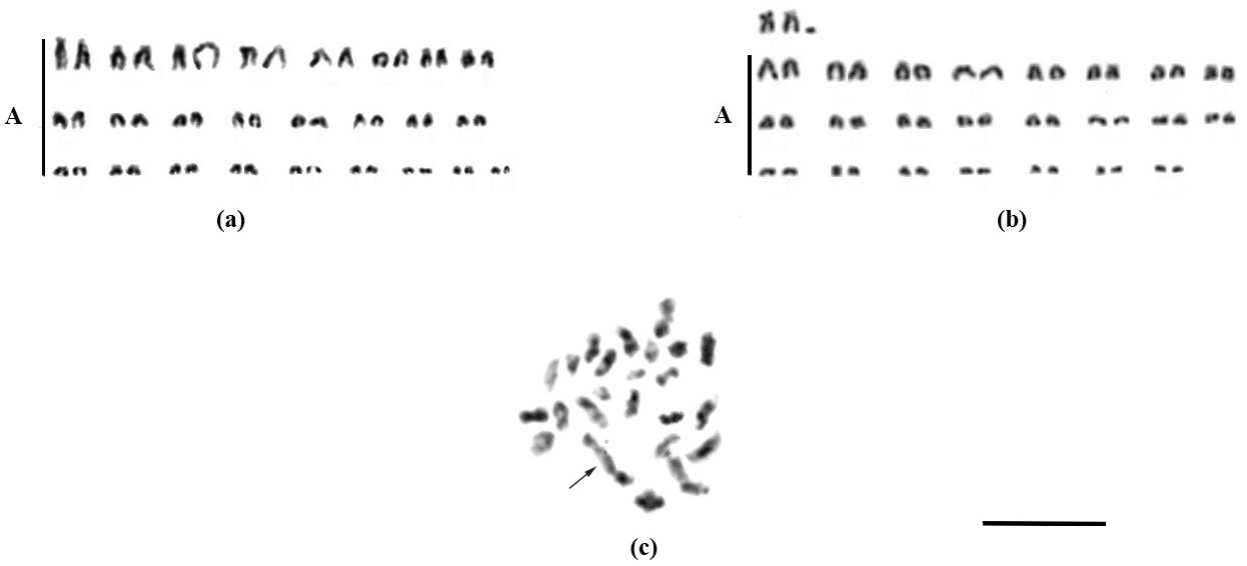
Karyotypes of *Nothobranchius brieni* **a** somatic chromosomes of female **b** somatic chromosomes of male **c** meiotic metaphase I (testicular). (A – acrocentric chromosomes). Note trivalent chromosome (arrowed). Scale bar: 10 µm.

The diploid chromosome numbers of *Nothobranchius brieni* were 2n = 49 for males and 2n = 50 for females with NF = 50/50 respectively. The female karyotype consisted of 25 pairs of acrocentric chromosomes gradually decreasing in size (Fig. 2a). The male karyotype consisted of 23 pairs of acrocentric chromosome and one bi-armed pair and two unpaired acrocentric chromosomes (Fig. 2b). In the first meiotic chromosomes during spermatogenesis 23 bivalents and a trivalent were observed at diakinesis (Fig. 2c).

**Figure 3.**
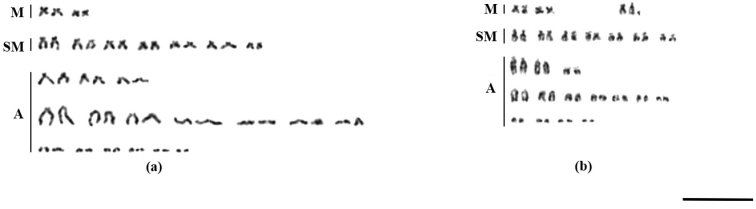
Karyotypes of *Nothobranchius* sp. ‘Kasenga’ **a** somatic chromosomes of female **b** somatic chromosomes of male (M – metacentric chromosomes, SM – submetacentric chromosomes, A – acrocentric chromosomes). Scale bar: 10 µm.

The karyotype *Nothobranchius* sp. ‘Kasenga’ had diploid number 2n = 49 for males and 2n = 50 for females with NF = 68/68 respectively. The female karyotype had two pairs of metacentric, seven pairs of sub-metacentric, and 16 pair of acrocentric chromosomes varying in size from large to small (Fig. 3a). The male karyotype had 23 pair of chromosomes similar to the female with one bi-armed and two unpaired acrocentric chromosomes (Fig. 3b).

## Discussion

### Cytogenetic characteristics

The described karyotypes stand apart from those already reported for species of genus *Nothobranchius*. The karyotype of *Nothobranchius brieni* has the chromosomal number 2n = 49/50 and 25 pairs of uni-armed chromosomes in female (50A) and 23 pairs of uni-armed homomorphic and three heteromorphic chromosomes in male (1M + 48A). The karyotype of *Nothobranchius* sp. ‘Kasenga’ has the same diploid number 2n = 49/50 but a different karyotype structure possessing metacentric, sub-metacentric, and uni-armed chromosomes with 4M + 14SM + 32A for females and 5M + 14SM + 30A for males, while other species of the genus have a considerably lower modal diploid number of only 36 chromosomes (Table 1).

**Table 1.** The diploid number (2n) of *Nothobranchius* species (from Arai 2011 with modifications). *sex chromosome system of the type X_1_X_2_Y/X_1_X_1_X_2_X_2_.

Species	2n	References
*Nothobranchius brieni* Poll, 1938*	49♂/50♀	Current study
*Nothobranchius eggersi* Seegers, 1982	36	Scheel 1990
*Nothobranchius elongatus* Wildekamp, 1982	38	Scheel 1990
*Nothobranchius foerschi* Wildekamp & Berkenkamp, 1979	34	Ewulonu et al. 1985, Scheel 1990
*Nothobranchius furzeri* Jubb, 1971	38	Reichwald et al. 2009
*Nothobranchius guentheri* (Pfeffer, 1893)*	35♂/36♀	Ewulonu et al. 1985, Scheel 1990
*Nothobranchius hengstleri* Valdesalici, 2007	38	Wildekamp et al. 2009
*Nothobranchius janpapi* Wildekamp, 1977	38	Scheel 1990
*Nothobranchius jubbi* Wildekamp & Berkenkamp, 1979	34	Scheel 1990
*Nothobranchius kirki* Jubb, 1969	36	Scheel 1990
*Nothobranchius korthausae* Meinken, 1973	36	Scheel 1990
*Nothobranchius krysanovi* Shidlovskiy, Watters & Wildekamp, 2010	18	Shidlovskiy et al. 2010
*Nothobranchius kuhntae* (Ahl, 1926)	38	Scheel 1990
*Nothobranchius lucius* Shidlovskiy, Watters & Wildekamp, 2010	36	Wildekamp et al. 2009
*Nothobranchius makondorum* Shidlovskiy, Watters & Wildekamp, 2010	38	Wildekamp et al. 2009
*Nothobranchius melanospilus* (Pfeffer, 1896)	38	Ewulonu et al. 1985
*Nothobranchius microlepis* (Vinciguerra, 1897)	24	Scheel 1990
*Nothobranchius palmqvisti* (Lönnberg, 1907)	36	Ewulonu et al. 1985, Scheel 1990
*Nothobranchius polli* Wildekamp, 1978	36	Ewulonu et al. 1985
*Nothobranchius patrizii* (Vinciguerra, 1897)	36	Ewulonu et al. 1985
*Nothobranchius pienaari* Shidlovskiy, Watters & Wildekamp, 2010	34	Shidlovskiy et al. 2010
*Nothobranchius rachovii* Ahl, 1926	16	Ewulonu et al. 1985, Krysanov 1992
*Nothobranchius steinforti* Wildekamp, 1977	36	Scheel 1990
*Nothobranchius thierryi* (Ahl, 1924)	43	Scheel 1990
*Nothobranchius* sp. ‘Kasenga’*	49♂/50♀	Current study

### Sex chromosomes

The reduced diploid numbers and heteromorphic chromosomes in males suggest the occurrence of a multiple-sex chromosome system. A trivalent observation in the first meiotic chromosomes in *Nothobranchius brieni* and the presence of a bi-armed chromosome exclusively in the male karyotype indicate a multiple-sex chromosome system of the type X_1_X_2_Y/X_1_X_1_X_2_X_2_. One bi-armed neo-Y chromosome has most likely resulted from the Robertsonian fusion between the Y chromosome and an autosome, as has been described for other fish species (e.g., Kitano and Peichel 2012). In *Nothobranchius brieni* and *Nothobranchius* sp. ‘Kasenga’ the Y chromosomes is a large metacentric one, and X_1_ and X_2_ chromosomes are acrocentric of different sizes. The same-sex chromosome system has been reported only for *Nothobranchius guentheri* (Ewulonu et al. 1985) among the 23 previously karyotyped species.

### Karyotype evolution

In the genus *Nothobranchius* and the related *Aphyosemyon* Mayers, 1924 the evolutionary trend to reduce the total number of chromosomes via acrocentric chromosome fusion was specified (Scheel 1990, Völker et al. 2005). This assumption has been confirmed by the data presented in Table 1. According to this hypothesis, basal taxa have higher chromosome numbers and more acrocentric chromosomes while derived taxa have lower numbers of chromosomes with metacentric chromosomes (Agnese et al. 2006). It is widely accepted that the hypothetical ancestral karyotype of teleostean fishes consisted of 2n = 48-50 acrocentric chromosomes (Ohno et al. 1969, Nakatani et al. 2007). The two species presented in this study have numbers and a structure of karyotype conservative for Actinopterygii fishes (Mank and Avise 2006, Molina et al. 2014). It is supposed that karyotype of *Nothobranchius brieni* is similar to that of the hypothetical ancestor of the genus *Nothobranchius*. There is a lack of molecular genetic data on this species, therefore we are not able to consider its phylogenetic position within the clade.
